# Dysregulation of NF-κB-Associated LncRNAs in Autism Spectrum Disorder

**DOI:** 10.3389/fnmol.2021.747785

**Published:** 2021-09-28

**Authors:** Kasra Honarmand Tamizkar, Elham Badrlou, Termeh Aslani, Serge Brand, Shahram Arsang-Jang, Soudeh Ghafouri-Fard, Mohammad Taheri

**Affiliations:** ^1^Department of Medical Genetics, School of Medicine, Shahid Beheshti University of Medical Sciences, Tehran, Iran; ^2^Department of Genetics, Tehran Medical Sciences Branch, Islamic Azad University, Tehran, Iran; ^3^Center for Affective, Stress and Sleep Research, Psychiatric Clinics, University of Basel, Basel, Switzerland; ^4^Division of Sport Science and Psychosocial Health, Department of Sport, Exercise and Health, University of Basel, Basel, Switzerland; ^5^Substance Abuse Prevention Research Center, Kermanshah University of Medical Sciences, Kermanshah, Iran; ^6^Sleep Disorder Research Center, Kermanshah University of Medical Sciences, Kermanshah, Iran; ^7^School of Medicine, Tehran University of Medical Sciences, Tehran, Iran; ^8^Cancer Gene therapy Research Center, Zanjan University of Medical Science, Zanjan, Iran; ^9^Men's Health and Reproductive Health Research Center, Shahid Beheshti University of Medical Sciences, Tehran, Iran; ^10^Skull Base Research Center, Loghman Hakim Hospital, Shahid Beheshti University of Medical Sciences, Tehran, Iran

**Keywords:** autism spectrum disorder, *ADINR*, *ANRIL*, *DILC*, *NKILA*, *CHAST*

## Abstract

Autism spectrum disorder (ASD) is a long-standing neurodevelopmental condition with prominent effects on social behavior of affected children. This disorder has been linked with neuroinflammatory responses. NF-κB has been shown to affect these responses in the orbitofrontal cortex of patients with ASD, thus being implicated in the pathogenesis of ASD. We measured expression of some NF-κB-associated lncRNAs and mRNAs (*DILC, ANRIL, PACER, CHAST, ADINR, DICER1-AS1, HNF1A-AS1, NKILA, ATG5* and *CEBPA*) in the peripheral blood of ASD kids vs. healthy children. Expression quantities of *ADINR, ANRIL, DILC, NKILA* and *CHAST* were meaningfully higher in ASD cases compared with healthy kids (Posterior Beta = 1.402, *P* value < 0.0001; Posterior Beta = 2.959, *P* value < 0.0001; Posterior Beta = 0.882, *P* value = 0.012; Posterior Beta = 1.461, *P* value < 0.0001; Posterior Beta = 0.541, *P* value = 0.043, respectively). The Bonferroni corrected *P* values for these lncRNAs remained significant except for *CHAST* and *DILC*. Expression levels of other genes were not considerably different between cases and controls. Expressions of *ATG5, DICER-AS1* and *DILC* were correlated with age of ASD patients (*P* < 0.0001). Among ASD cases, the most robust correlation has been detected between *ADINR* and *NKILA* (*r* = 0.87, *P* < 0.0001). Expression of none of genes has been correlated with age of healthy children. Among this group of children, expression levels of *ADINR* and *CHAST* were robustly correlated (*r* = 0.83, *P* < 0.0001). *ANRIL* had the greatest AUC value (AUC = 0.857), thus the best diagnostic power among the assessed genes. *NKILA* ranked the second position in this regard (AUC = 0.757). Thus, NF-κB-associated lncRNAs might partake in the pathogenesis of ASD.

## Introduction

Autism spectrum disorder (ASD) is a long-standing neurodevelopmental condition pigeonholed by defects in social abilities and speaking communication and the occurrence of stereotypic behaviors and interests (American Psychiatric Association, 2013). A wide range of genetic, environmental, neurological, and immune-related parameters partake in the etiology of ASD (Neuhaus et al., 2010). Growing evidence suggests the influence of inflammatory responses in the anterior areas of the neocortex in the pathophysiology of ASD (Pardo et al., 2005; Vargas et al., 2005). Moreover, stimulation of microglia and astrocytes in the brain regions correlated with cognitive activities has led to neuroinflammatory responses in these patients (Anderson et al., 2008). Nuclear factor κ-light-chain-enhancer of activated B cells (NF-κB) has been shown to regulate response to extracellular stress and expression amounts of pro-inflammatory cytokines (Pahl, 1999; Perkins, 2004). A previous study has reported aberrant levels of NF-κB in orbitofrontal cortex of patients with ASD especially in extremely activated microglia and its role in the molecular cascade resulting in the neuroinflammation, particularly in inhabitant immune cells in brain areas linked with the behavioral changes in ASD (Young et al., 2011). Moreover, expression of IKKα kinase which phosphorylates IκBα thus enhances the inhibitory impact of this factor on NF-κB, has been remarkably elevated in the cerebellum of patients with ASD. Besides, the expression of NF-κB and its phosphorylation at Ser536 have been considerably altered in the cerebellum and cortex of ASD patients and animal model of this disorder (Malik et al., 2011). We have newly identified dysregulation of some of the NF-κB-associated genes and lncRNAs in the peripheral blood of patients with schizophrenia vs. healthy controls (Safa et al., 2020a). In the current project, we measured expression of these lncRNAs and mRNAs (*DILC, ANRIL, PACER, CHAST, ADINR, DICER1-AS1, HNF1A-AS1, NKILA, ATG5* and *CEBPA*) in the peripheral blood of patients with ASD vs. healthy children. LncRNAs are a group of regulatory transcripts with sizes more than 200 nt to several kbs. These transcripts have influential effects on expression of genes through modulating chromatin structure, serving as enhancers for transcription, serving as molecular decoys to limit availability of other regulatory molecules and making scaf-folds for recruitment of other biomolecules (Fang and Fullwood, 2016). A bulk of evidence has shown particular involvement of these transcripts in the pathoetiology of neurodevelopmental diseases (Roberts et al., 2014).

## Materials and Methods

### ASD Patients and Normally Developed Children

The current research project was accomplished using the blood samples acquired from 30 ASD children (male/female ratio: 19/11) with mean age of 6 years (Standard deviation = 1.39). Forty-one age- and sex-matched healthy children (male/female ratio: 30/11) from the same ethnic group were selected as controls. ASD status was diagnosed by a psychiatric according to the DSM-V (American Psychiatric Association, 2013). Children having other neuropsychiatric, metabolic or immune-related conditions were omitted from the project. None of the ASD patients had a comorbid disorder such as attention deficit hyperactive disorder. Children registered as controls had no personal or familial history of neuropsychiatric disorders or developmental problem. Written informed consent forms were signed by all parents. The study protocol was approved by the ethical committee of Shahid Beheshti University of Medical Science and all methods were performed in accordance with the relevant guidelines and regulations (IR.SBMU.RETECH.REC.1399.740).

### Evaluation of Transcript Levels of Genes

RNA was retrieved from blood specimens using the extraction kit (Hybrid-R^TM^ Blood RNA) purchased from GeneAll Company (Seoul, South Korea). Next, 10 μg of extracted RNA specimens was converted to cDNA using the BioFact RT kit (Seoul, South Korea). Expression of genes was quantified in all specimens using the RealQ Plus 2x Master Mix (Amplicon, Denmark) and the primers which are shown in Table 1. All experiments were performed in duplicate. Each PCR run included a negative control (no template control) for each set of primers. Based on our previous studies validating the constant levels of *B2M* gene in the blood (Fallah et al., 2019), expression quantities of *B2M* gene were measured to normalize the expression data of target genes.

**Table 1:** Information about primers and the corresponding amplified region.

**Name**	**Type**	**Sequence**	**Primer Length**	**PCR Product Length**
NKILA-F	lncRNA	AACCAAACCTACCCACAACG	20	108
NKILA-R		ACCACTAAGTCAATCCCAGGTG	20	
ADINR-F	lncRNA	AGGGTGGATGTGCTGTGATGAAGA	24	98
ADINR-R		AGTCCATAACACCTCCGCAGACAA	24	
CEBPA-F	mRNA	ATTGCCTAGGAACACGAAGCACGA	24	161
CEBPA-R		TTTAGCAGAGACGCGCACATTCAC	24	
DICER1-AS1-F	lncRNA	CGAAGAAATGGAATAACTTCCAAC	24	125
DICER1-AS1-R		TTGGTCCAAACACAGAAGATC	21	
ATG5-F	mRNA	TTCGAGATGTGTGGTTTGGAC	21	134
ATG5-R		CACTTTGTCAGTTACCAACGTCA	23	
HNF1A-AS1-F	lncRNA	TCAAGAAATGGTGGCTAT	18	148
HNF1A-AS1-R		GCTCTGAGACTGGCTGAA	18	
CHAST-F	lncRNA	GCAGAGGGTGCCAACTTGTA	20	109
CHAST-R		TCTCAGGGAAATCAGATTGCGG	22	
B2M-F	mRNA	AGATGAGTATGCCTGCCGTG	20	105
B2M-R		GCGGCATCTTCAAACCTCCA	20	
ANRIL-F	lncRNA	TGCTCTATCCGCCAATCAGG	20	108
ANRIL-R		GCGTGCAGCGGTTTAGTTT	19	
DILC-F	lncRNA	GGAAAGGAGAGAAGAATGG	19	144
DILC-R		GTAAGATGTGGTTGTCGG	18	
PACER-F	lncRNA	TGGTCCTAAGCAGTTACCCTGTA	23	177
PACER-R		ACCAAAATAATCCACGCATCAGG	23	

### Statistical Strategies

Statistical methods were executed in Stan, “ggplot2,” “brms” and pROC packages in the R v.4 environment. The Spearman correlation coefficients were calculated to judge the correlation between expressions of the selected genes. Expression of these genes were compared between ASD children and normal children using the Bayesian regression model, the asymmetric Laplace family prior with 2,000 burnouts and 5,000 iterations. Rhat, posterior predictive plots and Loo were used. *P* values were corrected for multiple comparisons. To obtain the Bonferroni corrected *P* value, the original α-values were divided by the quantities of analyses on the dependent variable. Receiver operating characteristic (ROC) curves were illustrated to evaluate the diagnostic power of genes.

## Results

Expression quantities of *ADINR, ANRIL, DILC, NKILA* and *CHAST* were meaningfully higher in ASD cases compared with healthy kids (Posterior Beta = 1.402, *P* value < 0.0001; Posterior Beta = 2.959, *P* value < 0.0001; Posterior Beta = 0.882, *P* value = 0.012; Posterior Beta = 1.461, *P* value < 0.0001; Posterior Beta = 0.541, *P* value = 0.043, respectively). The Bonferroni corrected *P* values for these lncRNAs remained significant except for *CHAST* and *DILC*.

Expression levels of other genes were not considerably different between cases and controls. Table 2 reports the detailed parameters obtained from Bayesian regression model for evaluation of gene expression between ASD children and control subjects.

**Table 2:** Detailed parameters obtained from Bayesian regression model for comparison of gene expression between ASD children and control subjects (Expression Ratio: case/control; Gender: girl/boy; *P*-Value was calculated from Median regression model using bootstrap).

	**Posterior Beta of ER**	**SE**	***P*-Value**	**95% CrI for ER**
**CEBPA**				
Group	−0.122	0.56	0.409	[−1.21, 0.92]
Gender	−0.019	0.41	0.801	[−0.85, 0.77]
Age (>6 y to <6 y)	0.903	0.42	0.128	[0.1, 1.73]
**ADINR**				
Group	1.402	0.33	<0.0001	[0.73, 2.03]
Gender	−0.004	0.33	0.851	[−0.65, 0.61]
Age (>6 y to <6 y)	0.208	0.41	0.717	[−0.61, 1.04]
**ANRIL**				
Group	2.959	0.47	<0.0001	[2.07, 3.91]
Gender	0.35	0.45	0.53	[−0.5, 1.23]
Age (>6 y to <6 y)	−0.033	0.53	0.671	[−1.04, 1.03]
**ATG5**				
Group	−0.363	0.23	0.078	[−0.81, 0.08]
Gender	0.423	0.22	0.198	[−0.01, 0.84]
Age (>6 y to <6 y)	0.44	0.25	0.249	[−0.05, 0.95]
**DICER-AS1**				
Group	−2.076	0.44	0.603	[−2.87, −1.13]
Gender	0.149	0.43	0.568	[−0.7, 0.98]
Age (>6 y to <6 y)	0.102	0.43	0.519	[−0.65, 1]
**DILC**				
Group	0.882	0.32	0.012	[0.23, 1.5]
Gender	0.146	0.34	0.737	[−0.53, 0.84]
Age (>6 y to <6 y)	−0.726	0.61	0.702	[−1.95, 0.44]
**HNF1A-AS1**				
Group	0.578	0.43	0.177	[−0.2, 1.47]
Gender	−0.129	0.45	0.732	[−1.07, 0.7]
Age (>6 y to <6 y)	−0.071	0.49	0.743	[−1.06, 0.9]
**NKILA**				
Group	1.461	0.36	<0.0001	[0.79, 2.18]
Gender	0.112	0.39	0.536	[−0.69, 0.85]
Age (>6 y to <6 y)	0.246	0.47	0.983	[−0.74, 1.1]
**PACER**				
Group	−0.013	0.46	0.805	[−0.96, 0.89]
Gender	−0.197	0.44	0.461	[−1.05, 0.61]
Age (>6 y to <6 y)	−0.128	0.47	0.738	[−1.06, 0.78]
**CHAST**				
Group	0.541	0.27	0.043	[0.003, 1.09]
Gender	0.132	0.3	0.788	[−0.46, 0.73]
Age (>6 y to <6 y)	0.216	0.33	0.449	[−0.39, 0.9]

**Figure 1:**
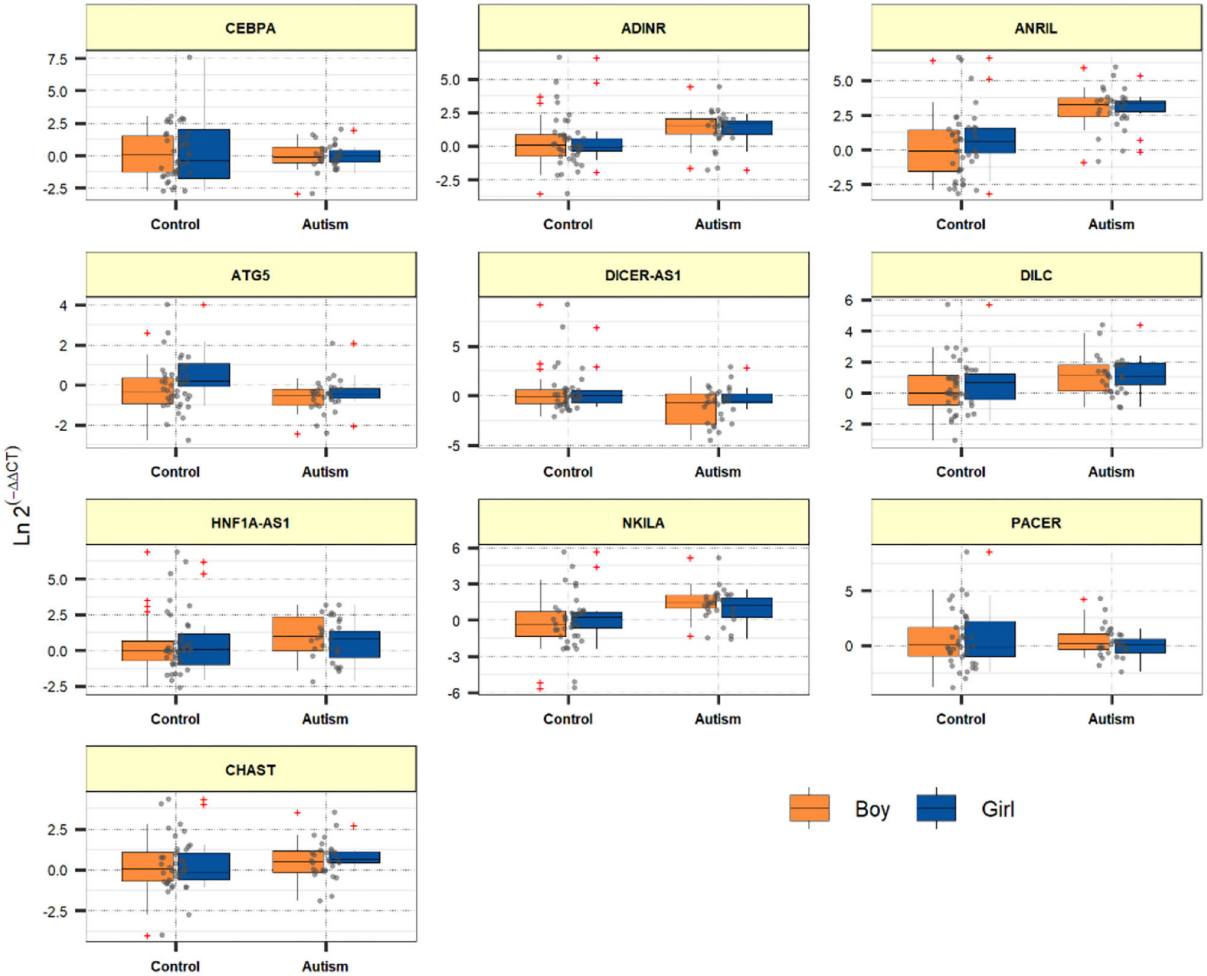
Relative levels of assessed genes among ASD children and healthy children.

Figure 1 demonstrates the levels of assessed genes among ASD children and normal children.

### Correlation Between Expressions of Genes

**Figure 2:**
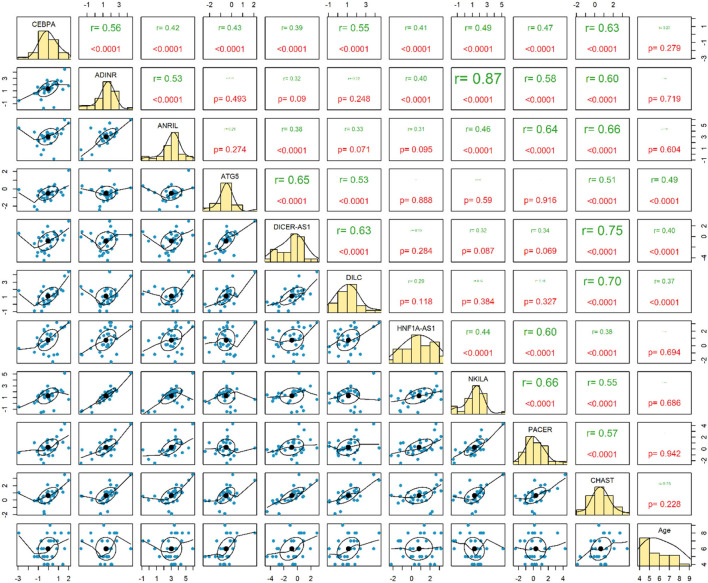
Correlation between expressions of genes among patients with ASD.

Expressions of *ATG5, DICER-AS1* and *DILC* were correlated with age of ASD patients (*P* < 0.0001). Among ASD cases, the most considerable correlation has been detected between *ADINR* and *NKILA* (*r* = 0.87, *P* < 0.0001) (Figure 2).

**Figure 3:**
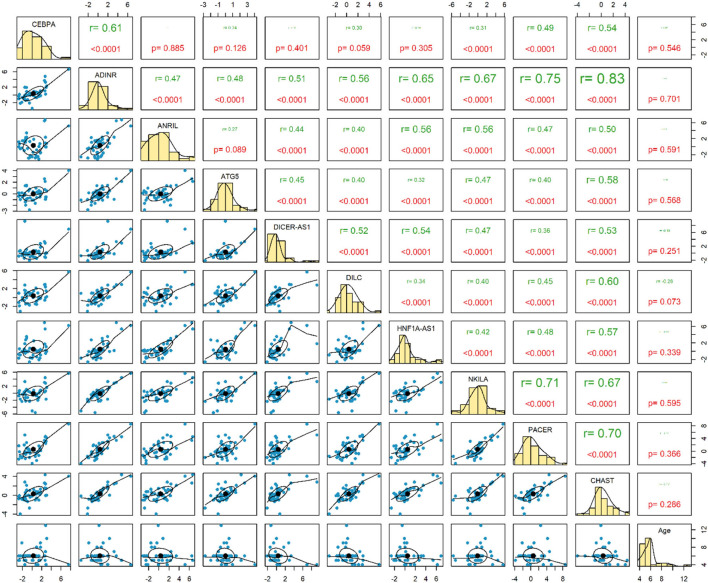
Correlation between expressions of genes among healthy controls.

Expression of none of genes has been correlated with age of healthy children. Among this group of children, expression levels of *ADINR* and *CHAST* were robustly correlated (*r* = 0.83, *P* < 0.0001) (Figure 3).

### ROC Curves

**Figure 4:**
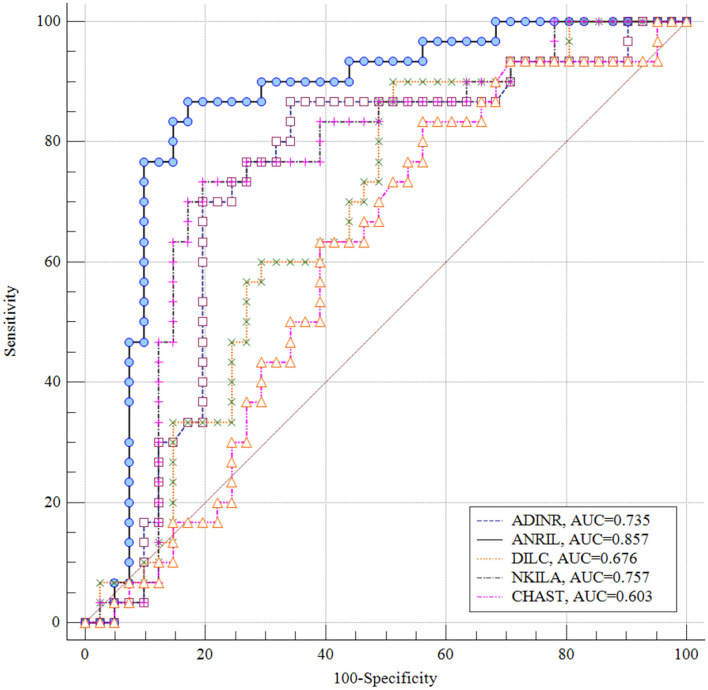
Receiver Operating Characteristics (ROC) curves for appraisal of diagnostic value of *ADINR, ANRIL, DILC, NKILA* and *CHAST* in ASD.

*ANRIL* had the greatest AUC value (AUC = 0.857), thus the best diagnostic power among the assessed genes. *NKILA* ranked the second position in this regard (AUC = 0.757). Figure 4 displays the plotted ROC curves for distinguishing ASD from healthy status.

## Discussion

NF-κB has an essential role in the regulation of immune responses which partake in the pathogenesis of ASD. Based on our recent experience in patients with schizophrenia regarding the possible role of NF-κB-related genes, we hypothesized that these genes might also be involved in ASD. This hypothesis has been supported by the existence of inflammatory responses at the cross point of shared underlying mechanisms for schizophrenia and autism (Meyer et al., 2011). Notably, we detected over-expression of *ADINR, ANRIL, DILC, NKILA* and *CHAST* in the peripheral blood of ASD cases compared with controls. However, the Bonferroni corrected *P* values for *CHAST* and *DILC* did not remain significant. *ADINR* is an lncRNA which is transcribed from a region upstream of the *CEBPA* gene, regulating its expression. This lncRNA binds with PA1 and recruits histone methyl-transferase complexes to alter histone epigenetic marks in the *CEBPA* locus (Xiao et al., 2015). Although the role of CEBPA in the pathogenesis of ASD has not been elucidated yet, CEBPA has functional interactions with the ASD-related protein CHD8 (Kita et al., 2018). Moreover, CEBPA has been shown to modulate nfkb1 expression and relocate histone deacetylases from NF-κB p50 homodimers to activate expression of NF-κB target genes (Paz-Priel et al., 2011). Therefore, *ADINR* might participate in the pathogenesis of ASD through modulating CEBPA or CHD8 activity or expression levels. We have recently demonstrated a trend toward association between some *ANRIL* haplotypes and risk of ASD among Iranians (Safa et al., 2020b). Moreover, *ANRIL* has an acknowledged role in the regulation of immune responses as an element of NF-κB pathway (Zhou et al., 2016). DILC has an appreciated role in the pathogenesis a the immune-mediated disorder rheumatoid arthritis through modulating IL-6 expression (Wang et al., 2019). IL-6 is possibly involved in the pathogenesis of ASD since its expression has been elevated IL-6 is increased in the cerebellum of patients with ASD and it changes adhesion and migration of neurons and modulates development of synapses (Wei et al., 2011). *NKILA* has been shown to decline TNF-α-associated inflammatory responses (Han et al., 2020). Notably, cytokine profiling has recognized TNF-α as an important dysregulated cytokine in patients with ASD (Xie et al., 2017). Finally, *CHAST* over-expression has also been detected in patients with schizophrenia (Safa et al., 2020a). Expression levels of other genes were not considerably different between cases and controls.

It is worth mentioning that levels of lncRNAs circulating in peripheral blood do not necessarily correlate with their levels in the brain. Since brain tissues are not available from these patients, we could not evaluate correlations between these two sets of samples.

Notably, expressions of *ATG5, DICER-AS1* and *DILC* were correlated with age of ASD patients, but not healthy subjects, indicating different impact of age-related factors among patients and healthy controls. Previous studies have reported age-dependent changes in genes expressions in the brain tissues of ASD cases. For instance, Chow et al. have shown altered expressions of pathways controlling cell number, cortical patterning, and differentiation in prefrontal cortex samples of young ASD cases. On the other hand, signaling and repair pathways have been found to be dysregulated in prefrontal cortex samples of adult ASD cases. They have concluded that age-dependent alterations in gene signature in ASD might represent distinctive abnormal processes in different phases of neurodevelopment (Chow et al., 2012). Moreover, experiments in lymphoblastoid cell lines have shown an obvious inconsistency between neuroanatomical and cellular aberrations detected in ASD cases at younger ages and molecular abnormalities at higher age (Ansel et al., 2017). Thus, identification of age-related changes in gene expression might facilitate recognition of the most important biomedical pathways in pathoetiology of ASD in each age.

The most robust correlations have been detected between *ADINR* and *NKILA* among cases, and between *ADINR* and *CHAST* among healthy persons, indicating the impact of ASD on modulation of the correlation between NF-κB-associated genes.

Finally, we appraised the suitability of genes in distinguishing between ASD and healthy status. *ANRIL* had the greatest AUC value, thus the best diagnostic power among the assessed genes. *NKILA* ranked the second position in this regard. Thus, NF-κB-associated lncRNAs might partake in the pathogenesis of ASD and can be used as diagnostic markers in ASD. However, the AUC data need to be replicated in a larger sample in which ASD diagnosis is established using a standardized diagnostic tool such as the ADOS or ADI-R before it can be clinically useful. Moreover, functional experiments are desired to appraise the molecular cascade of involvement of NF-κB-associated lncRNAs in ASD.

## Data Availability Statement

The original contributions presented in the study are included in the article/supplementary files, further inquiries can be directed to the corresponding authors.

## Ethics Statement

The studies involving human participants were reviewed and approved by the Ethical Committee of Shahid Beheshti University of Medical Science and all methods were performed in accordance with the relevant guidelines and regulations (IR.SBMU.RETECH.REC.1399.740). Written informed consent to participate in this study was provided by the participants' legal guardian/next of kin.

## Author Contributions

EB, TA, and MT performed the experiment. SA-J analyzed the data. SG-F and KH designed and supervised the study. SG-F wrote and revised the draft. All the authors are contributed equally and approved the submitted version.

## Funding

The current study was supported by a grant from Shahid Beheshti University of Medical Sciences.

## Conflict of Interest

The authors declare that the research was conducted in the absence of any commercial or financial relationships that could be construed as a potential conflict of interest.

## Publisher's Note

All claims expressed in this article are solely those of the authors and do not necessarily represent those of their affiliated organizations, or those of the publisher, the editors and the reviewers. Any product that may be evaluated in this article, or claim that may be made by its manufacturer, is not guaranteed or endorsed by the publisher.
